# Using linkage logic theory to control dynamics of a gene regulatory network of a chordate embryo

**DOI:** 10.1038/s41598-021-83045-y

**Published:** 2021-02-17

**Authors:** Kenji Kobayashi, Kazuki Maeda, Miki Tokuoka, Atsushi Mochizuki, Yutaka Satou

**Affiliations:** 1 Department of Zoology, Graduate School of Science, Kyoto University, Sakyo, Kyoto, 606-8502 Japan; 2 Faculty of Informatics, The University of Fukuchiyama, 3370 Hori, Fukuchiyama, Kyoto 620-0886 Japan; 3 Institute for Frontier Life and Medical Sciences, Kyoto University, Sakyo, Kyoto 606-8507 Japan

**Keywords:** Regulatory networks, Reprogramming

## Abstract

Linkage logic theory provides a mathematical criterion to control network dynamics by manipulating activities of a subset of network nodes, which are collectively called a feedback vertex set (FVS). Because many biological functions emerge from dynamics of biological networks, this theory provides a promising tool for controlling biological functions. By manipulating the activity of FVS molecules identified in a gene regulatory network (GRN) for fate specification of seven tissues in ascidian embryos, we previously succeeded in reproducing six of the seven cell types. Simultaneously, we discovered that the experimentally reconstituted GRN lacked information sufficient to reproduce muscle cells. Here, we utilized linkage logic theory as a tool to find missing edges in the GRN. Then, we identified a FVS from an updated version of the GRN and confirmed that manipulating the activity of this FVS was sufficient to induce all seven cell types, even in a multi-cellular environment. Thus, linkage logic theory provides tools to find missing edges in experimentally reconstituted networks, to determine whether reconstituted networks contain sufficient information to fulfil expected functions, and to reprogram cell fate.

## Introduction

Animal embryos differentiate a variety of cell types, each of which expresses a specific set of genes. Such specific gene expression patterns emerge from dynamics of gene regulatory networks (GRNs)^1^. In GRNs, nodes represent genes, and edges represent regulatory interactions between nodes. Regulatory genes that encode transcription factors and signaling molecules regulate one another, and constitute the core of GRNs. Non-regulatory genes, which do not regulate others and have only input edges, are located at the periphery of GRNs.

Given mathematical formulae of regulatory functions for all nodes in a network, we can determine dynamics of a GRN precisely. However, from a practical standpoint, it is difficult to identify regulatory functions, because gene expression is controlled by complex mechanisms, usually depending on activities of multiple factors. On the other hand, mathematical theories have been developed to control dynamics of network systems using structural information, without assuming regulatory functions or related parameters^2–4^. Among these mathematical theories, one called linkage logic is promising, because it is applicable to linear- and non-linear dynamics. This theory claims that dynamics of a network system with multiple steady states (attractors) can be controlled by manipulating activities of a set of key nodes, which are determined only from structural information. These key nodes are collectively called a feedback vertex set (FVS).

In a previous study^5^, we applied this theory to a GRN that specifies cell fates in embryos of an ascidian (*Ciona intestinalis* Type A or *Ciona robusta*), an invertebrate animal belonging to the sister group of vertebrates. The GRN structure has been determined by comprehensive knock-down assays for regulatory genes that are expressed during embryogenesis^6,7^. GRN dynamics produce multiple steady states that evoke gene expression patterns specific for individual cell types. Specifically, by the gastrula stage, the ascidian GRN specifies seven major cell types, although some of these differentiate further into subtypes thereafter^6,7^. Linkage logic theory has identified five nodes, *Foxa.a*, *Foxd*, *Neurog*, *Zic-r.b*, and the Erk signaling pathway as a FVS^5^. An exhaustive test, in which each of these five factors was up- or down-regulated, succeeded in reproducing six cell types (epidermis, brain, neural cells other than brain, notochord, mesenchyme, and endoderm); thus, it largely substantiated the theory. This study showed that qualitative up- and down-regulation are sufficient to control dynamics of the GRN in early ascidian embryos. At the same time, it suggested the possibility that the GRN structure used did not contain sufficient information for muscle fate specification. In the present study, we applied linkage logic theory to identify missing edges that were not included in the experimentally determined GRN structure.

In our previous study^5^, to accurately reproduce a system that the theory assumes, we adopted artifactual syncytium embryos, in which no intercellular interactions are expected. However, because cell types are often maintained cell-autonomously, embryonic cells may be controllable through this theory in a multi-cellular condition. To extend the application of this theory, we also tried to substantiate the theory in a multi-cellular condition.

## Results

### Identification of candidates for missing edges

Linkage logic theory claims that GRN dynamics are controllable by manipulating activities of FVS molecules. On the other hand, in our previous exhaustive examination of the GRN specifying cell fates in ascidian embryos in binary space (up- and down-regulation of FVS molecules), muscle cells were not produced. Therefore, the GRN structure, which had been determined by comprehensive knock-down assays for regulatory genes^6,7^ (Table S1), apparently lacked nodes (regulatory molecules) or edges (regulatory interactions), and previously identified FVSs may consequently have lacked one or more nodes. Because zygotic expression of almost all regulatory genes has been examined in early ascidian embryos by in situ hybridization^8^, it was less likely that there were missing nodes. For this reason, we thought that perhaps the GRN structure lacked important edges that were components of a subnetwork responsible for muscle fate specification, such that one or more FVS nodes were not identified.

**Figure 1:**
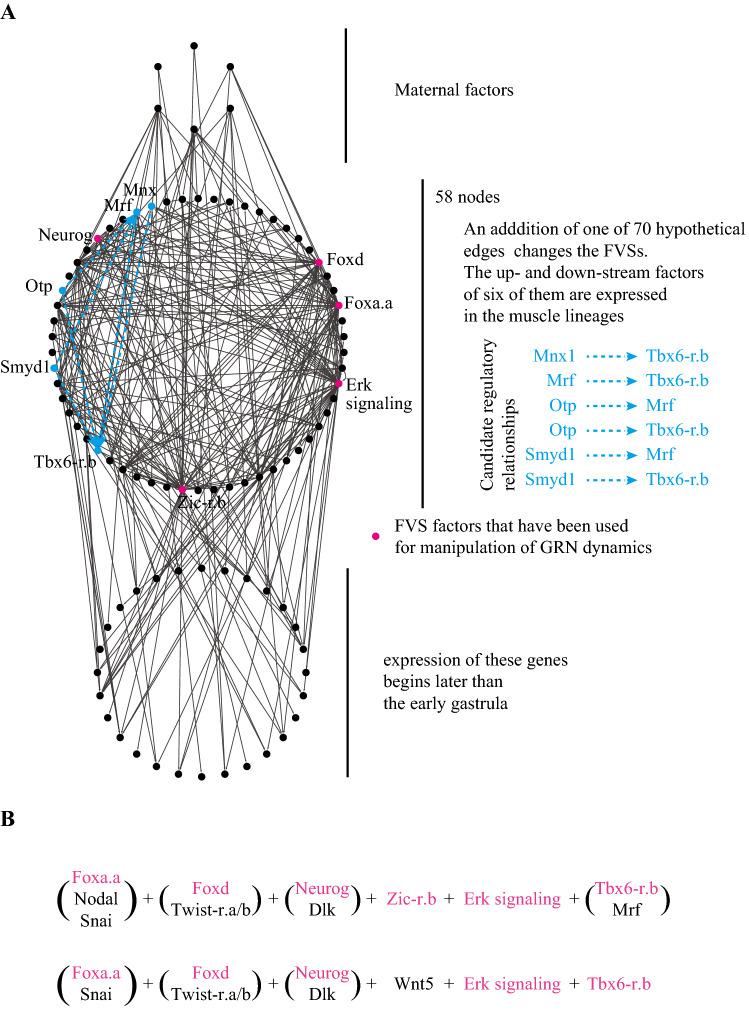
The gene regulatory network for cell fate specification in ascidian embryos. (**A**) The GRN consists of three layers, maternal factors, zygotically expressed factors during fate specification, and genes expressed later. Nodes and edges are represented by dots and arrows. The second layer consists of 58 nodes. An exhaustive search for hypothetical edges that change FVSs identified 70 edges. Among them, edges for which upstream and downstream factors are expressed in muscle cells are shown in cyan. FVS molecules used for manipulation of GRN dynamics are indicated by magenta dots. (**B**) FVSs identified in the updated version of the GRN. Parentheses indicate multiple choices; therefore, there are 32 different FVSs. Factors we used for controlling cell fate are shown in magenta.

To identify such edges, we exhaustively repeated theoretical tests. In each test, we added one hypothetical edge between two nodes, and examined whether the network with this hypothetical edge had FVSs different from the original FVSs. We excluded maternal factors, because they are not regulated by other factors. We also excluded genes that begin to be expressed later than the early gastrula stage, because cell fate specification occurs by that stage. Indeed, although FGF treatment converts presumptive muscle cells to mesenchymal cells, their competence is lost before early gastrula stage^9^, suggesting that muscle fate is specified before early gastrula stage. As a result, with 58 regulators and 191 known interactions, we tested 3115 (= 58 × 57–191) hypothetical edges. In these theoretical tests, we found that 70 hypothetical edges (regulatory interactions) changed FVSs. Upstream and/or downstream factors of six of these hypothetical edges are expressed in the muscle lineage; therefore, they became the primary candidates (Fig. 1A).

A recent study has indeed confirmed one of the predicted edges in which Mrf regulates *Tbx6-r.b*^10^. The network with this edge had 32 FVSs, each consisting of six nodes. Because it does not have FVSs consisting of ≤ five nodes, these 32 FVSs are minimal. These minimal FVSs were {Foxa.a|Nodal|Snai, Foxd|Twist-r.a/b, Neurog|Dlk, Zic-r.b, Erk signaling, Tbx6-r.b|Mrf}6 or {Foxa.a |Snai, Foxd|Twist-r.a/b, Neurog|Dlk, Wnt5, Erk signaling, Tbx6-r.b}, where ‘‘|’’ indicates a multiple choice (3 × 2  × 2 × 1 × 1 × 2 + 2 × 2 × 2 × 1 × 1 × 1 = 32 sets; see Fig. 1B).

### Controlling dynamics of the GRN that specifies cell fate

Two of the newly identified FVSs contained *Foxa.a*, *Foxd*, *Neurog*, *Zic-r.b*, and the Erk signaling pathway, which were nodes that we used to control GRN dynamics in our previous study^5^, as well as an additional node, *Tbx6-r.b* or *Mrf*. We chose the set consisting of *Foxa.a*, *Foxd*, *Neurog*, *Zic-r.b*, *Tbx6-r.b*, and the Erk signaling pathway for the following analyses.

**Figure 2:**
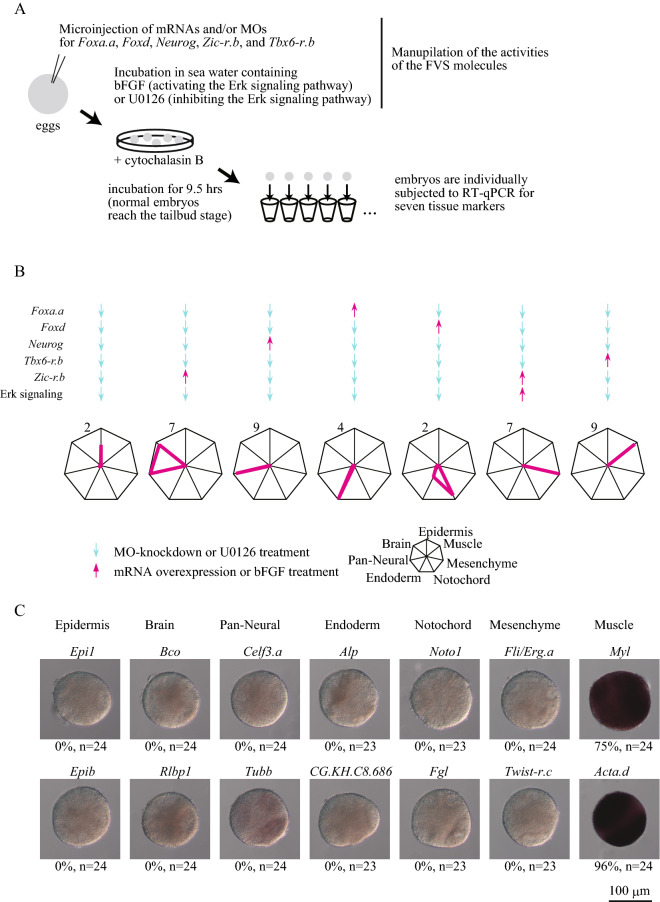
Seven different cell types are reproduced by manipulating activities of FVS factors in single-cell syncytium embryos. (**A**) The experimental design. (**B**) FVS factors are up- or down-regulated in seven different combinations, as shown at the top. Radar graphs show expression levels of marker genes relative to their corresponding values in normal 9.5-h (tailbud) embryos in the seven experimental conditions. Each axis shows expression of a tissue marker as shown at the bottom. Mean values for 12 embryos from two batches (six from each) are shown. Values for individual embryos are shown in Fig. 3. Note that brain cells express the brain and pan-neural markers. (**C**) In situ hybridization of single-cell syncytium embryos in which *Tbx6-r.b* was overexpressed and the remaining FVS factors were down-regulated. Only muscle marker genes were expressed. Percentages that expressed the indicated markers and numbers of embryos we examined are shown beneath the photographs. Marker genes that were also used in the RT-qPCR assay shown in (**B**) are shown in the upper row.

We first used an experimental system for single-cell development that we used in our previous study^5^, because it was not necessary to consider intercellular interactions in this system, which was close to the condition assumed by the theory (Fig. 2A). For this purpose, we incubated fertilized eggs in sea water containing cytochalasin B, which inhibits cytokinesis. In cells treated with this drug, nuclear divisions continue, and specification dynamics proceed^11–21^. At 9.5 h post-fertilization, when normal embryos express tissue marker genes, expression of marker genes was examined by reverse-transcription, followed by quantitative PCR (RT-qPCR) for 12 embryos from two different batches (six from each batch). Marker genes were the same as those used in our previous study^5^: *Epi1* for epidermis, *Bco* for the brain, *Celf3.a* for the entire neural system, *Alkaline phosphatase* (*Alp*) for endoderm, *Noto1* for the notochord, *Fli/Erg.a* for mesenchyme, and *Myosin light chain* (*Myl*) for muscle^8,22–30^.

**Figure 3:**
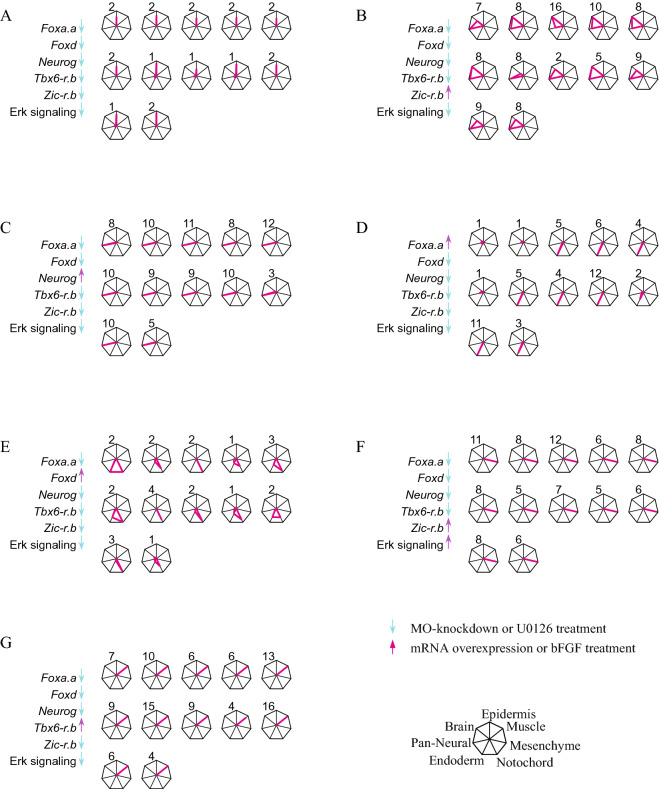
Gene expression in individual embryos in which cell fate is reprogrammed by manipulating activities of FVS factors in single-cell syncytium embryos. FVS factors are up- or down-regulated in seven different combinations as shown at the left of each panel. Radar graphs show expression levels of marker genes relative to their corresponding values in normal 9.5-h (tailbud) embryos. Each axis shows expression of a tissue marker, as shown at the bottom right. Values for twelve individual embryos, used for calculation of mean values in Fig. 2B, are shown in each condition. Note that brain cells express the brain and pan-neural markers.

On the basis of results in our previous study^5^, we first tried to produce the six cell types that we produced previously. When we down-regulated activities of all six molecules (knockdown of *Foxa.a*, *Foxd*, *Neurog*, *Zic-r.b*, and *Tbx6-r.b* using morpholino antisense oligonucleotides and treatment with the MEK inhibitor, U0126, which downregulates ERK activity), an epidermal marker was predominantly expressed (mean values for 12 embryos are shown in Fig. 2B, and expression levels for individual embryos are shown in Fig. 3A). Similarly, expression patterns of these marker genes indicated that brain, other neural tissues, notochord, mesenchyme, and endoderm fates were specified under conditions shown in Fig. 2B. These results were reproduced in almost all experimental embryos (Fig. 3B–F). Finally, when *Tbx6-r.b* was overexpressed and the remaining FVS factors were downregulated, a muscle marker was specifically expressed in all embryos (Figs. 2B, 3G). Using in situ hybridization for the above and additional marker genes (*Epib* for epidermis, *Rlbp1* for the brain, *Tubb* for the entire neural system, *CG.KH.C8.686* for endoderm, *Fgl* for the notochord, *Twist-r.c* for mesenchyme, and *Acta.d* for muscle), we further confirmed that this manipulation specified muscle fate uniquely (Fig. 2C). Specifically, no marker genes other than the muscle markers, *Myl* and *Actin* (*Acta.d*), were expressed in embryos in which *Tbx6-r.b* was overexpressed and the remaining FVS factors were downregulated. Thus, we succeeded in obtaining all seven expected cell types by manipulating activities of the FVS factors identified from the updated version of the GRN structure.

### Manipulating activities of FVS factors under a multi-cellular condition

Each cell has its own GRN and these GRNs are interconnected through cell–cell interactions. In other words, GRNs that govern gene expression in individual cells are subnetworks of a larger GRN that governs gene expression within an embryo. However, result with the single-cell syncytium system indicated that by manipulating activities of the FVS factors, intercellular interactions are not necessary to produce the seven cell types. For this reason, we expected the above seven cell types to be produced by the same manipulation under multi-cellular conditions. This is consistent with the notion that cells determined for a specific fate cannot easily be transformed^31^.

**Figure 4:**
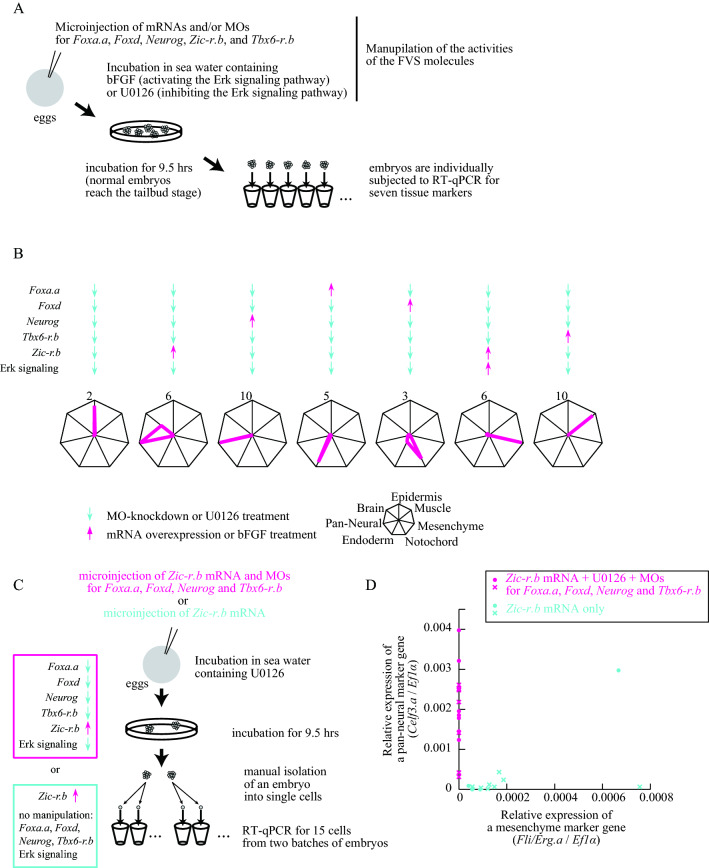
Reprograming of cell fate by manipulating activities of FVS factors under a multi-cellular condition. (**A**) The experimental design for cell fate control under a multi-cellular condition. (**B**) FVS factors are up- or down-regulated in seven different combinations as shown at the top. Radar graphs show expression levels of marker genes relative to their corresponding values in normal 9.5-h (tailbud) embryos in the seven experimental conditions. Each axis shows expression of a tissue marker, as shown at the bottom. Mean values for 12 embryos from two batches (six from each) are shown. Values for individual embryos are shown in Fig. 5. (**C**,**D**) Relative expression levels of the pan-neural (*Celf3.a*) and mesenchyme (*Fli/Erg.a*) markers to the expression level of *Ef1α* in individual cells in two conditions (magenta and cyan). The experimental design is shown in (**C**). In (**D**), cells from different batches are shown by circles and crosses.

**Figure 5:**
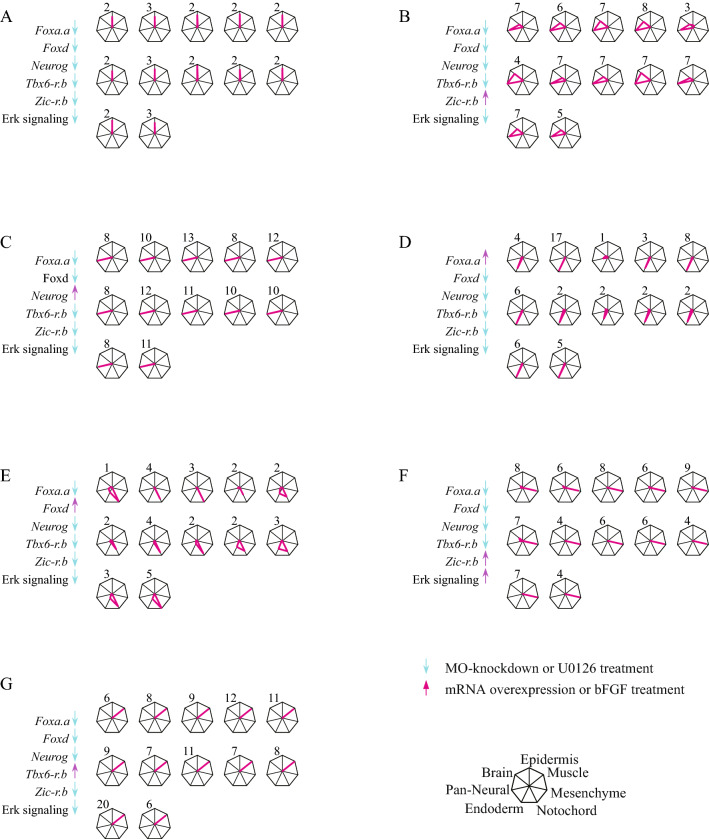
Gene expression in individual embryos in which cell fate is reprogrammed by manipulating activities of FVS factors under a multi-cellular condition. FVS factors are up- or down-regulated in seven different combinations, as shown at the left of each panel. Radar graphs show expression levels of marker genes relative to their corresponding values in normal 9.5-h (tailbud) embryos. Each axis shows expression of a tissue marker, as shown at the bottom right. Values for twelve individual embryos, used for calculation of the mean values in Fig. 4B, are shown in each condition. Note that brain cells express the brain and pan-neural markers.

We therefore examined whether the same experimental manipulations produce the seven cell types when cytochalasin B was not added (Fig. 4A). Marker genes were specifically expressed by manipulating activities of the FVS factors in the same way as in the single-cell syncytium experimental system (Figs. 4B, 5). Thus, manipulation of FVS activities drove GRN dynamics deterministically into a single-cell state.

To confirm that all cells have a single cell fate in such experimental embryos, we examined expression of marker genes at single-cell resolution using embryos in which *Zic-r.b* was upregulated and the remaining FVS factors were downregulated. After a 9.5-h incubation, we dissociated embryos into single cells, and picked 10 cells from each of two batches of embryos (Fig. 4C). We analyzed these 20 cells individually by RT-qPCR. Among them, we excluded 5 cells from which we failed to obtain sufficient amplification of a ubiquitously expressed gene, *Ef1α.* We detected expression of the pan-neural marker, *Celf3.a*, in the remaining 15 cells. As a negative control, we also measured expression of the mesenchyme marker, *Fli/Erg.a*, and as expected, no amplification was detected (Fig. 4D). This observation strongly suggests that all cells in the above experimental embryos express the neural marker specifically.

Similarly, we injected only *Zic-r.b* mRNA alone and examined gene expression in 13 individual cells from two batches of embryos. While we detected *Celf3.a* expression in three cells, we detected *Fli/Erg.a* expression in all cells including the three cells with *Celf3.a* expression (Fig. 4C,D). In other words, overexpression of *Zic-r.b* alone did not drive cells into neural fate deterministically. That is, these two experiments indicated that not only overexpression of *Zic-r.b* but also downregulation of activities of the remaining FVS factors are necessary to drive cells into neural fate. In addition, the latter experiment showed that overexpression of *Zic-r.b* alone cannot determine cell fate uniquely, which is consistent with a proposition of linkage logic theory that network dynamics are fully controllable by manipulation of all FVS factors.

## Discussion

Linkage logic theory was developed to control network dynamics by manipulating activities of a small subset of nodes. Because key nodes are identified from network structure only, it is applicable to a wide range of linear or non-linear networks, and provides a powerful tool for controlling network dynamics. In the present study, we applied linkage logic theory to the updated version of the *Ciona* GRN, and succeeded in inducing all of the expected cell states by controlling activities of the FVS molecules.

The GRN structure that we used in our previous study^5^ lacked an important edge, which represented activation of *Tbx6-r.b* by *Mrf*. Consequently, the GRN structure did not contain sufficient information for muscle fate specification. Cell fates other than muscle were induced by conditions in which *Tbx6-r.b* was downregulated in the present study, while we never observed expression of a muscle marker by manipulating activities of the five FVS factors other than *Tbx6-r.b* in our previous study^5^. This may indicate that *Tbx6-r.b* expression was suppressed under conditions in which activities of the five FVS factors other than *Tbx6-r.b* were manipulated.

It is possible that the developmental program specifying the muscle fate was not turned on in the single-cell syncytium system. A maternal factor localized at the posterior pole is essential for specification of the muscle fate^32,33^. Because this maternal factor works together with other maternal factors to activate downstream genes^34^, it may not be able to activate downstream genes necessary for specification of the muscle fate in the single-cell syncytium system. Thus, it is not inconsistent with the theory that six cell types other than muscle cells were induced by manipulation of activities of the five FVS factors. On the other hand, the present study showed that Tbx6-r.b is necessary to induce the muscle fate, and manipulation of activities of the entire FVS, including Tbx6-r.b, is necessary to completely control GRN dynamics.

Experiments in the present study were performed in a binary manner (i. e. up- or down-regulation of the FVS factors). Our success in producing all seven expected cell types may indicate that quantitative regulation is not important for the GRN to specify cell fate in ascidian embryos. This property may not be specific to ascidian embryos, because the endomesodermal GRN in early sea urchin embryos is represented by Boolean functions^35^.

On the other hand, we observed a low level of expression of the endoderm marker in embryos that predominantly expressed the notochord maker in several embryos (Figs. 3E, 5E). It is possible that more precise quantitative manipulation is necessary to reduce such noisy expression of the endoderm marker.

Structures of biological networks are generally determined by functional experiments, in which activities of individual nodes are up- or down-regulated, and/or inferred from high-throughput analysis data, including RNA-seq and ChIP-seq (chromatin-immunoprecipitation followed by deep-sequencing) analyses. Precise reconstruction of biological networks is laborious, and it is often difficult to determine whether reconstructed networks have enough information to fulfil expected functions. Linkage logic theory mathematically assures that FVSs are minimally sufficient subsets of genes to reproduce all possible attractors of the system by manipulating their behavior. Therefore, if not all expected states are obtained by exhaustive manipulation of FVS activities, the network structure from which the FVS is deduced must be inaccurate. That is, this can be a criterion to determine whether the network structure contains sufficient information to fulfil expected functions. The *Ciona* GRN for fate specification satisfies this criterion.

If a reconstructed GRN does not satisfy the above criterion, it lacks important nodes or edges. While missing nodes (regulatory genes) can easily be identified through high-throughput expression assays, missing edges cannot be. This is because edges represent regulatory interactions; therefore, functional experiments are necessary to identify such interactions. As we showed, linkage logic theory is useful for finding an important edge that the reconstructed network lacked, but the real network has. That is, hypothetical edges that change FVSs are strong candidates.

Specifically, if a reconstituted regulatory network is likely to lack an important edge for reproducing dynamics, we can efficiently screen candidates by examining all possible edges (regulatory interactions). These possible edges are added to the network one by one, and computational tests are performed to choose hypothetical edges that change FVSs from the original FVSs. After this screening, each candidate should be examined experimentally. This experimental test determines which candidate edges really exist and whether network dynamics are fully controllable by manipulating activities of newly identified FVS factors. Although it is possible to test networks by adding two or more hypothetical edges, we succeeded in completely controlling the network dynamics to induce all expected cell types by adding only one.

Linkage logic theory determines important node sets from the network structure alone, without assuming mathematical formulae of regulatory functions or related parameters^2–4^. Therefore, it provides two strong predictions: (1) a criterion to determine whether the network structure contains sufficient information to fulfil expected functions, and (2) candidate missing edges if the network structure does not contain sufficient information. On the other hand, it does not predict how gene expression patterns change with time. For such a prediction, model-based approaches, which commonly assume formulae of regulatory functions and/or tuning parameter values to explain observed gene expression patterns, are required^35,36^. Thus, model-free linkage logic theory and model-based approaches are complementary.

In animal development, cells gradually lose pluripotency, and are finally specified to a particular cell type. In specified cells, cell-type-specific differentiation programs are induced, and differentiated cells generally maintain their states cell-autonomously. Therefore, it is not surprising that manipulation of activities of the FVS of the GRN for fate-specification changes all cells in an embryo into one specific cell type. However, this will not always be true for GRNs that fulfil other functions. Some cells may maintain their cell states through interactions with different cell types.

## Methods

### Animals, whole-mount in situ hybridization, and gene identifiers

Adult *Ciona intestinalis* (type A; also called *Ciona robusta*) were obtained from the National Bio-Resource Project for *Ciona* in Japan. cDNA clones were obtained from our EST clone collection^37^. Whole-mount in situ hybridization was performed as described previously^38^. Gene identifiers, according to the nomenclature rule^39–41^, are shown in Table S2.

### Identification of FVSs using linkage logic theory

The mathematical background for linkage logic theory and its application to gene regulatory networks have been described previously^2,4,5^.

Gene activities in a GRN, which is represented by a directed graph $\Gamma = \left( {V,E} \right)$ ($V$, a node or gene set; $E$, an edge or regulatory linkage set), are modelled by a system of ordinary differential equations. Under the assumption that gene activities, measured in terms of concentrations of mRNAs or proteins, decay in the absence of supply or synthesis, dynamics of activity $x_{n}$ of gene $n{ } \in V$ are written in the form:

1 $$\dot{x}_{n} = F\left( {\varvec{x}} \right) = F_{n} \left( {x_{n} ,{\varvec{x}}_{{I_{n} }} } \right)$$

with the ‘decay condition’:

2 $$\partial_{1} F_{n} \left( {x_{n} ,{\varvec{x}}_{{I_{n} }} } \right) < 0$$

The set $I_{n} \subseteq V$ is the input set of $n$, a subset of molecules that regulate molecule $n$, that is, $I_{n} = \left\{ {i|\left( {i \to n} \right) \in E} \right\}$. The notation $\partial_{1}$ implies the first partial derivative with respect to the first argument. The set $I_{n}$ includes $n$ ($n \in I_{n}$) if and only if the dynamics of gene $n$ regulate self-activation. Even if $\partial F_{n} /\partial x_{n}$ is not negative, the decay condition (2) can be satisfied by adding a hypothetical decay term and a compensating positive term indicating a self-regulatory loop. The sets of $I_{n}$ ($\forall n{ } \in V$) directly represent the graphical structure of the regulatory network. Under formulations (1) and (2), we proved that sets of key nodes for dynamics are determined from the topology of the network as FVSs^2,4,42^. In graph theory, an FVS is defined as a subset of vertices in a directed graph, the removal of which leaves a graph without directed cycles^43^. The theorem implies that (1) by observing dynamical behaviors of a FVS of a network, we should be able to identify all asymptotic behaviors of dynamics of the whole network system, and (2) by controlling behaviors of a FVS of a network, we should be able to control the whole system to converge to any asymptotic behaviors of the system.

The GRN we used for identification of FVSs is shown in Table S1 and summarized in Fig. 1A. We first identified nodes (genes) that are not regulated by others and nodes that do not regulate other nodes, and repeatedly removed these nodes and connecting edges from the gene regulatory network, because removal of these nodes does not affect identification of FVSs. Then, we applied the depth-first search algorithm^5^ and identified 32 FVSs that contained six nodes.

### Gene knockdown and overexpression

All morpholino antisense oligonucleotides (MOs) (Gene Tools, LLC) used in the present study block translation. These MOs have been used previously and their specificity has been evaluated^6,10,44–48^; *Foxa.a* MO, 5′-ATCCGATTTCAAAAGCTTTCTCAGA-3′; *Foxd* MO, 5′-GCACACAACACTGCACTGTCATCAT-3′; *Neurog* MO, 5′-AAATCCAACATTTTGTAGCAAGAGC-3′; *Tbx6-r.b* MO, 5′-TTACAATTTCCTCTCTCTTTCGATT-3′; *Zic-r.b*, 5′-GATCAACCATTACATTAGAATACAT-3′. For synthetic mRNAs, coding sequences of *Foxa.a*, *Foxd*, *Neurog*, *Tbx6-r.b,* and *Zic-r.b* were cloned into pBluescript RN3^49^, and synthetic mRNAs were transcribed using the mMESSAGE mMACHINE T3 Transcription Kit (Thermo Fisher Scientific). Each of the MOs was prepared at a concentration of 0.3 mM, and each mRNA was prepared at a concentration of 0.5 μg/μL for *Foxa.a*, *Foxd*, *Neurog*, and *Zic-r.b* or 0.05 μg/μL for *Tbx6-r.b*. Mixtures of MOs and mRNAs were injected into eggs in a volume of 30 pL. Injection of a control MO against *E. coli lacZ* (5′-TACGCTTCTTCTTTGGAGCAGTCAT-3′) at a concentration of 1.5 mM or control *lacZ* mRNA at a concentration of 2 μg/μL yielded larvae with normal morphology. To arrest cell division, embryos were incubated in seawater containing 2.5 μg/mL cytochalasin B (Sigma). For up- and down-regulation of Erk signaling, we treated embryos with 10 ng/mL human recombinant basic FGF (Sigma, F0291) and 2 μM of the MEK inhibitor U0126 (Calbiochem).

Reverse transcription followed by quantitative PCR (Figs. 2, 3, 4A,B, and 5) was performed using a Cells-to-CT kit (Thermo Fisher Scientific). Each experimental embryo was placed in a tube and reverse-transcription was performed in accordance with the manufacturer’s instructions. Single-cell-reverse transcription followed by quantitative PCR (Fig. 4C,D) was performed using a Single Cell-to-CT Kit (Thermo Fisher Scientific). Dissociation of manipulated early tailbud embryos was performed as described previously^50^. Single cells were placed in tubes and reverse-transcription and pre-amplification were performed in accordance with the manufacturer’s instructions. Quantitative PCR was performed using the TaqMan method with primers and probes shown in Table S3.

## Supplementary Information

Supplementary Information.
