# Supplementary material for: Using linkage logic theory to control dynamics of a gene regulatory network of a chordate embryo
Source: Sci Rep. 2021 Feb 17;11:4001. doi: 10.1038/s41598-021-83045-y (PMC7889898; doi:10.1038/s41598-021-83045-y)
Supplement: Supplementary file 1 — Supplementary Information. [file 41598_2021_83045_MOESM1_ESM.pdf]

Supplementary Tables S1 to S3 for

Using linkage logic theory to control dynamics of a gene regulatory network of a chordate embryo

Kenji Kobayashi[1], Kazuki Maeda[2], Miki Tokuoka[1], Atsushi Mochizuki[3], and Yutaka Satou[1]

1. Department of Zoology, Graduate School of Science, Kyoto University, Sakyo, Kyoto, 606-8502, Japan
2. Faculty of Informatics, The University of Fukuchiyama, 3370 Hori, Fukuchiyama, Kyoto 620-0886, Japan
3. Institute for Frontier Life and Medical Sciences, Kyoto University, Sakyo, Kyoto 606-8507, Japan

*Authors for correspondence:

Atsushi Mochizuki (mochi@infront.kyoto-u.ac.jp)

Yutaka Satou (yutaka@ascidian.zool.kyoto-u.ac.jp)

Table S1. Regulatory interactions in the developmental gene regulatory network in *Ciona* embryos up to the late gastrula stage.

Upstream factor	Downstream factor	activation/repression
Admp	Admp	repression
Admp	Fgf8/17/18	activation
Admp	Nodal	repression
Admp	Otx	repression
Admp	Tbx2/3	activation
Bmp2/4	Bmp2/4	repression
Bmp2/4	Tbx2/3	activation
Brachyury	Brachyury	repression
Brachyury	Fgl**	repression
Brachyury	Noto1**	repression
Ctnnb*	Alp**	activation
Ctnnb*	Efna.d	repression
Ctnnb*	Fgf9/16/20	activation
Ctnnb*	Foxa.a	activation
Ctnnb*	Foxd	activation
Ctnnb*	Lhx3/4	activation
Ctnnb*	Tbx6-r.b	activation
Ctnnb*	Tfap2-r.b	repression
Ctnnb*	Zic-r.b	repression
Ctnnb*	Zic-r.b	activation
Dlk	Brachyury	activation
Dlk	Cdx**	activation
Dlk	Dlk	repression
Dlk	Ebf3**	activation
Dlk	Efna.d	activation
Dlk	Fgf8/17/18	repression
Dlk	Foxb	repression
Dlk	Gsx**	activation
Dlk	Hes.b	activation
Dlk	Id	activation
Dlk	Meis**	repression

Dlk	Mrf	activation
Dlk	Neurog	activation
Dlk	Pax6**	activation
Dlx.b	Dlx.b	repression
Dlx.b	Emx**	activation
Dlx.b	Epi1**	activation
Dlx.b	Epib**	activation
Dlx.b	Foxc	activation
Dlx.b	Foxh.a**	activation
Dlx.b	Gata.b	activation
Dlx.b	Myt1	activation
Dlx.b	Six3/6**	activation
Dlx.b	Zic-r.b	activation
Dmrt.a	Dmrt.a	repression
Dmrt.a	Foxc	activation
Dmrt.a	Six3/6**	activation
Efna.d	Erk signaling	repression
Erk signaling	BHLHA15**	activation
Erk signaling	Brachyury	activation
Erk signaling	Cers.e**	activation
Erk signaling	Chd	activation
Erk signaling	Dlk	activation
Erk signaling	Dmrt.a	activation
Erk signaling	Dusp1/2/4/5	activation
Erk signaling	Ebf3**	activation
Erk signaling	Efna.c	activation
Erk signaling	Elk	activation
Erk signaling	Epi1**	repression
Erk signaling	Fgf8/17/18	activation
Erk signaling	Fgf8/17/18	repression
Erk signaling	Fgf9/16/20	repression
Erk signaling	Fli/Erg.a	activation
Erk signaling	Fos	activation
Erk signaling	Foxb	repression
Erk signaling	Foxc	repression

Erk signaling	Foxc	activation
Erk signaling	Hhex**	activation
Erk signaling	Id	activation
Erk signaling	Jun	activation
Erk signaling	Lhx3/4	activation
Erk signaling	Mesp	activation
Erk signaling	Mnx1	activation
Erk signaling	Mrf	repression
Erk signaling	Msx	activation
Erk signaling	Myt1	activation
Erk signaling	Nkx2-1/4	activation
Erk signaling	Nodal	activation
Erk signaling	Nog	activation
Erk signaling	Otx	activation
Erk signaling	Pax3/7**	activation
Erk signaling	Pax6**	activation
Erk signaling	Tfap2-r.b	repression
Erk signaling	Twist-r.a/b	activation
Erk signaling	Twist-r.c**	activation
Erk signaling	Zf249	activation
Erk signaling	Zic-r.b	activation
Fgf8/17/18	Erk signaling	activation
Fgf9/16/20	Erk signaling	activation
Foxa.a	Alp**	activation
Foxa.a	Brachyury	activation
Foxa.a	CG.KH2012.C8.686**	activation
Foxa.a	Chd	activation
Foxa.a	Dlk	activation
Foxa.a	Dlk	repression
Foxa.a	Dmrt.a	activation
Foxa.a	Dusp1/2/4/5	activation
Foxa.a	Emx**	repression
Foxa.a	Eph.a	activation
Foxa.a	Foxb	activation
Foxa.a	Foxc	activation

Foxa.a	Fzd4	activation
Foxa.a	Gata.b	activation
Foxa.a	Hand-r	activation
Foxa.a	Lhx3/4	activation
Foxa.a	Mnx1	activation
Foxa.a	Msx	repression
Foxa.a	Myt1	activation
Foxa.a	Nkx2-1/4	activation
Foxa.a	Nodal	repression
Foxa.a	Otx	activation
Foxa.a	Pax3/7**	repression
Foxa.a	Pax6**	activation
Foxa.a	Sfrp1/5	activation
Foxa.a	Twist-r.a/b	activation
Foxa.a	Zf266**	activation
Foxa.a	Zic-r.b	activation
Foxb	Cdx**	repression
Foxb	Fgf8/17/18	repression
Foxb	Foxb	repression
Foxb	Mnx1	activation
Foxb	Pax6**	repression
Foxc	Foxc	repression
Foxc	Zic-r.b	repression
Foxd	Brachyury	activation
Foxd	Chd	activation
Foxd	Dlk	activation
Foxd	Dlx.b	repression
Foxd	Dmrt.a	repression
Foxd	Ebf3**	activation
Foxd	Fgf8/17/18	activation
Foxd	Fgf9/16/20	activation
Foxd	Foxa.a	activation
Foxd	Foxb	activation
Foxd	Foxd	repression
Foxd	Hand-r	activation

Foxd	Lhx3/4	activation
Foxd	Mnx1	activation
Foxd	Myt1	activation
Foxd	Neurog	activation
Foxd	Nkx2-1/4	activation
Foxd	Nodal	repression
Foxd	Otx	repression
Foxd	Pax6**	activation
Foxd	Prdm1-r.a	repression
Foxd	Prdm1-r.b	repression
Foxd	Twist-r.a/b	activation
Foxd	Twist-r.a/b	repression
Foxd	Wnt5	activation
Foxd	Zf266**	activation
Foxd	Zic-r.b	activation
Gata.a*	Efna.d	activation
Gata.a*	Epi1**	activation
Gata.a*	Epib**	activation
Gata.a*	Otx	activation
Gata.a*	Tfap2-r.b	activation
Gata.a*	Zic-r.b	activation
Gdf1/3-r	Nodal	repression
Gdf1/3-r	Otx	repression
Hand-r	Hand-r	repression
Hand-r	Twist-r.a/b	activation
Hes.a	Foxa.a	repression
Hes.a	Zic-r.b	repression
Id	Id	repression
Lefty	Lefty	repression
Lhx3/4	Alp**	activation
Lhx3/4	Mesp	activation
Lmx1	Lmx1**	repression
Mesp	Mesp	repression
Mrf	Acta1**	activation
Mrf	Mrf	repression

Mrf	Myl**	activation
Mrf	Otp	activation
Mrf	Smyd1	activation
Mrf	Tbx6-r.b	activation***
Msx	Lmx1**	activation
Neurog	Dlk	activation
Neurog	Ebf3**	activation
Neurog	Fgf8/17/18	activation
Neurog	Fgf8/17/18	repression
Neurog	Id	activation
Neurog	Myt1	activation
Neurog	Neurog	repression
Neurog	Snai	activation
Nodal	Chd	activation
Nodal	Dlk	activation
Nodal	Ebf3**	activation
Nodal	Efna.b**	activation
Nodal	Fgf8/17/18	activation
Nodal	Fgf9/16/20	repression
Nodal	Foxc	repression
Nodal	Hand-r	activation
Nodal	Hes.b	activation
Nodal	Id	activation
Nodal	Lefty	activation
Nodal	Lmx1**	activation
Nodal	Mrf	activation
Nodal	Msx	activation
Nodal	Myt1	repression
Nodal	Neurog	activation
Nodal	Nodal	repression
Nodal	Pax3/7**	activation
Nodal	Pax6**	activation
Nodal	Snai	activation
Nodal	Tbx6-r.b	activation
Nodal	Twist-r.a/b	activation

Otx	BHLHA15**	activation
Otx	Cers.e**	activation
Otx	Fli/Erg.a	activation
Otx	Fos	activation
Otx	Foxc	activation
Otx	Hhex**	activation
Otx	Jun	activation
Otx	Msx	activation
Otx	Myt1	activation
Otx	Otx	repression
Otx	Six3/6**	activation
Otx	Twist-r.a/b	activation
Otx	Twist-r.c**	activation
Pax3/7	Pax3/7**	repression
Pax6**	Pax6**	repression
Pem1*	Admp	repression
Pem1*	Fgf9/16/20	repression
Pem1*	Foxa.a	repression
Pem1*	Foxb	repression
Pem1*	Sox1/2/3	repression
Prdm1-r.a	Efna.d	activation
Prdm1-r.a	Foxa.a	repression
Prdm1-r.a	Prdm1-r.a	repression
Prdm1-r.a	Prdm1-r.b	repression
Prdm1-r.a	Six3/6**	repression
Prdm1-r.a	Zic-r.b	repression
Prdm1-r.b	Efna.d	activation
Prdm1-r.b	Six3/6**	repression
Prdm1-r.b	Zic-r.b	repression
Snai	Brachyury	repression
Snai	Efna.b**	activation
Snai	Efna.d	activation
Snai	Fgf8/17/18	repression
Snai	Foxa.a	repression
Snai	Gsx**	repression

Snai	Mnx1	repression
Snai	Myt1	repression
Snai	Snai	repression
Sox1/2/3	Dlx.b	activation
Sox1/2/3	Epib**	activation
Sox1/2/3	Foxc	activation
Sox1/2/3	Msx	activation
Sox1/2/3	Nodal	activation
Sox1/2/3	Otx	activation
Sox1/2/3	Six3/6**	activation
Sox1/2/3	Sox1/2/3	repression
Sox1/2/3	Zic-r.b	activation
Sox4/11/12	Cdx**	repression
Sox4/11/12	Dlk	repression
Sox4/11/12	Dmrt.a	activation
Sox4/11/12	Fgf8/17/18	repression
Sox4/11/12	Foxc	activation
Sox4/11/12	Gata.b	activation
Sox4/11/12	Myt1	activation
Sox4/11/12	Neurog	repression
Sox4/11/12	Nodal	repression
Sox4/11/12	Pax6**	repression
Sox4/11/12	Snai	repression
Tbx2/3	Msx	activation
Tbx2/3	Tbx2/3	repression
Tbx6-r.b	Mesp	activation
Tbx6-r.b	Mnx1	activation
Tbx6-r.b	Mrf	activation
Tbx6-r.b	Otp	activation
Tbx6-r.b	Smyd1	activation
Tbx6-r.b	Snai	activation
Tbx6-r.b	Tbx6-r.b	repression
Tbx6-r.b	Zic-r.b	activation
Tfap2-r.b	Epib**	activation
Tfap2-r.b	Id	repression

Tp53.a*	Brachyury	activation
Tp53.a*	Zic-r.b	activation
Tp53.b*	Brachyury	activation
Tp53.b*	Zic-r.b	activation
Twist-r.a/b	BHLHA15**	activation
Twist-r.a/b	Cers.e**	activation
Twist-r.a/b	Fli/Erg.a	activation
Twist-r.a/b	Fos	activation
Twist-r.a/b	Foxd	activation
Twist-r.a/b	Hhex**	activation
Twist-r.a/b	Twist-r.c**	activation
Wnt5	Wnt5	repression
Wnt5	Zic-r.b	activation
Wnttun5	Nodal	repression
Wnttun5	Otx	repression
Zic-r.a*	Admp	activation
Zic-r.a*	Hes.b	activation
Zic-r.a*	Lefty	activation
Zic-r.a*	Nodal	activation
Zic-r.a*	Otx	activation
Zic-r.a*	Snai	activation
Zic-r.a*	Tbx6-r.a	activation
Zic-r.a*	Tbx6-r.b	activation
Zic-r.a*	Wnt5	activation
Zic-r.a*	Wnttun5	activation
Zic-r.b	Bco**	activation
Zic-r.b	Brachyury	activation
Zic-r.b	Cdx**	activation
Zic-r.b	Celf3.a**	activation
Zic-r.b	Chd	activation
Zic-r.b	Dlk	activation
Zic-r.b	Ebf3**	activation
Zic-r.b	Efna.d	repression
Zic-r.b	Fos	activation
Zic-r.b	Lhx3/4	activation

Zic-r.b	Lmx1**	activation
Zic-r.b	Mnx1	activation
Zic-r.b	Mrf	activation
Zic-r.b	Myt1	activation
Zic-r.b	Neurog	activation
Zic-r.b	Otp	activation
Zic-r.b	Pax6**	activation
Zic-r.b	Rlbp1**	activation
Zic-r.b	Six3/6**	activation
Zic-r.b	Smyd1	activation
Zic-r.b	Snai	activation
Zic-r.b	Tbx6-r.a	activation
Zic-r.b	Tbx6-r.b	activation
Zic-r.b	Tubb**	activation
Zic-r.b	Twist-r.a/b	activation
Zic-r.b	Wnt5	activation
Zic-r.b	Zf266**	activation
Zic-r.b	Zic-r.b	repression

* Maternal factors

** Genes that begin to be expressed after fate specification

*** An edge recently identified

Table S2. Gene identifiers for genes used in the present study.

Gene	Identifier (CG.KH2012)	Identifier (CG.KY)
Acta.d	KH.C1.570	KY.Chr1.1965
Admp	KH.C2.421	KY.Chr2.773
Alp	KH.L153.31	KY.Chr6.211
Bco	KH.C9.224	KY.Chr9.917
BHLHA15	KH.C3.308	KY.Chr3.1309
Bmp2/4	KH.C4.125	KY.Chr4.449
Brachyury	KH.S1404.1	KY.Chr12.6
Cdx	KH.C14.408	KY.Chr14.625
Celf3.a	KH.C6.128	KY.Chr6.59
Cers.e	KH.C3.255	KY.Chr3.1370
CG.KH2012.C8.686	KH.C8.686	KY.Chr8.1063
Chd (*Chordin*)	KH.C6.145	KY.Chr6.371
Ctnnb (*β-catenin*)	KH.C9.53	KY.Chr9.48
Dlk (*Delta.b*)	KH.L50.6	KY.Chr11.146
Dlx.b	KH.L57.25	KY.Chr7.359
Dmrt.a	KH.S544.3	KY.Chr5.698
Dusp1/2/4/5	KH.C1.1079	KY.Chr1.1703
Ebf3	KH.L24.10	KY.Chr1.724
Eef1a1 (*Ef1a*)	KH.C14.52	KY.Chr14.194
Efna.b	KH.C3.202	KY.Chr3.888
Efna.c	KH.C3.52	KY.Chr3.891
Efna.d	KH.C3.716	KY.Chr3.893
Elk	KH.C8.247	KY.Chr8.613
Emx	KH.L142.14	KY.Chr8.1337
Eph.a	KH.C1.404	KY.Chr1.238
Epi1	KH.C1.188	KY.Chr1.2380
Epib	KH.C7.154	KY.Chr7.872
Fgf8/17/18	KH.C5.5	KY.Chr5.496
Fgf9/16/20	KH.C2.125	KY.Chr2.1217
Fgl	KH.C1.832	KY.Chr1.592
Fli/Erg.a	KH.C4.539	KY.Chr4.259
Fos	KH.C11.314	KY.Chr11.1133

Foxa.a	KH.C11.313	KY.Chr11.1167
Foxb	KH.C4.341	KY.Chr4.915
Foxc	KH.L57.25	KY.Chr12.156
Foxd	KH.C8.890/C8.396	KY.Chr8.660/661
Foxh.a	KH.C9.717	KY.Chr9.672
Fzd4	KH.C6.162	KY.Chr6.48
Gata.a	KH.L20.1	KY.Chr6.625
Gata.b	KH.S696.1	KY.Chr4.1344
Gdf1/3-r	KH.C4.547	KY.Chr4.450
Gsx	KH.C2.917	KY.Chr2.1386
Hand-r	KH.C1.1116	KY.Chr1.2070
Hes.a	KH.C1.159	KY.Chr1.28
Hes.b	KH.C3.312	KY.Chr3.580
Hhex	KH.L171.10	KY.Chr1.1187
Id	KH.C7.692/C7.157	KY.Chr7.1153/1157
Jun	KH.C5.610	KY.Chr5.536
Lefty	KH.C3.411	KY.Chr3.1493
Lhx3/4	KH.S215.4	KY.Chr13.449
Lmx1	KH.C9.616	KY.Chr9.589
Meis	KH.C10.174	KY.Chr10.902
Mesp	KH.C3.100	KY.Chr3.993
Mnx1	KH.L128.12	KY.Chr10.622
Mrf	KH.C14.307	KY.Chr14.1058
Msx	KH.L57.25	KY.Chr2.1417
Myl	KH.C1.1186/C1.20	KY.Chr1.2010/2011
Myt1	KH.C1.274	KY.Chr1.1536
Neurog	KH.C6.129	KY.Chr6.427
Nkx2-1/4	KH.C10.338	KY.Chr10.964
Nodal	KH.L106.16	KY.Chr14.1181
Nog (*Noggin*)	KH.C12.562	KY.Chr12.723
Noto1	KH.L20.18	KY.Chr6.606
Otp	KH.C14.377	KY.Chr14.946
Otx	KH.C4.84	KY.Chr4.867
Pax3/7	KH.C10.150	KY.Chr10.303
Pax6	KH.C9.68	KY.Chr9.1034

Pem1	KH.C1.755	KY.Chr1.944
Prdm1-r.a	KH.C12.493	KY.Chr12.980
Prdm1-r.b	KH.C12.105	KY.Chr12.977
Rlbp1	KH.C11.439	KY.Chr11.799
Sfrp1/5	KH.L171.5	KY.Chr1.1207
Six3/6	KH.C10.367	KY.Chr10.279
Smyd1	KH.S423.6	KY.Chr6.594
Snai (*Snail*)	KH.C3.751	KY.Chr3.1382
Sox1/2/3	KH.C1.99	KY.Chr1.559
Sox4/11/12	KH.C7.523	KY.Chr7.659
Tbx2/3	KH.L96.87	KY.Chr4.1104
Tbx6-r.a	KH.L8.11	KY.Chr11.460
Tbx6-r.b	KH.S654.3	KY.Chr11.470
Tfap2-r.b	KH.C7.43	KY.Chr7.1173
Tp53.a	KH.C1.573	KY.Chr1.1942
Tp53.b	KH.C3.713	KY.Chr3.226
Tubb	KH.L116.85	KY.Chr11.636
Twist-r.a/b	KH.C5.416/C5.554	KY.Chr5.355/356
Twist-r.c	KH.C5.202	KY.Chr5.357
Wnt5	KH.L152.45	KY.Chr4.1328
Wnttun5	KH.C9.257	KY.Chr9.807
Zf249	KH.C4.182	KY.Chr4.13
Zf266	KH.C1.777	KY.Chr1.2461
Zic-r.a (*Macho-1*)	KH.C1.727	KY.Chr1.1698
Zic-r.b (*ZicL*)	KH.L59.12/L59.1/S816.1/S816.4	KY.Chr6.26/27/28/29/30/31

Table S3. Probes and primers used for quantitative PCR.

Gene	Fluorescent Probe (5' to 3')	Forward primer (5' to 3')	Reverse primer (5' to 3')
Epi1	(FAM)-ATCCTCGATATGAATGCGGTTTCCCC-(TAMRA)	CCAGACAATGGTGTTGGAAGAC	AACGCAGTGGAATTGAGTCACA
Bco	(VIC)-TCAGATCGATCCGGTGACCCTTGATACA-(TAMRA)	TCGCCATCACTGAAAGCAACT	GTGTTTCGCAAGATCAACCTTGT
Celf3.a	(FAM)-CTCGCCAGTAGCACGAACGCCC-(TAMRA)	GGCAAACCAACTGCAAACAA	CAACCATCAGGCCCTTCTTTT
Alp	(FAM)-AATCCTATTTTCGGCGCCGCTCC-(TAMRA)	CGGATCACAGCCATGTTTTTAC	CGACGAGCTTTGGATTATTAACGT
Noto1	(VIC)-CGTTCATGTACGGGTTTCTTGCAACCA-(TAMRA)	GGCTTGCCTGCGAATGG	GAGCACACGACTGCATCGTAA
Fli/Erg.a	(FAM)-ACGAGAAGGCGACCACCAATACACGA-(TAMRA)	TCCTACTACAGGGCAGGAAGCT	ACCCAAAGTATGCAACGTGTTTT

Myl	(VIC)-CGAGCCATTAACCTTAACCCAACCATTGAA-(TAMRA)	TGGATTCGATCAAGTAGGAGATGTT	CAATTTTTTGGCAGCCATATCTT
Ef1a	(FAM)-CAAGAACATGATCACAGGAACATCCCAGG-(TAMRA)	CTCCCGGTCACAGAGATTTCA	CAATAAGCACGGCACAATCG
